# Global MLST of *Salmonella* Typhi Revisited in Post-genomic Era: Genetic Conservation, Population Structure, and Comparative Genomics of Rare Sequence Types

**DOI:** 10.3389/fmicb.2016.00270

**Published:** 2016-03-02

**Authors:** Kien-Pong Yap, Wing S. Ho, Han M. Gan, Lay C. Chai, Kwai L. Thong

**Affiliations:** ^1^Institute of Biological Sciences, Faculty of Science, University of MalayaKuala Lumpur, Malaysia; ^2^School of Science, Monash University MalaysiaBandar Sunway, Malaysia

**Keywords:** MLST, phylogenomic, *Salmonella* Typhi, sequence types, typhoid, whole genome sequencing

## Abstract

Typhoid fever, caused by *Salmonella enterica* serovar Typhi, remains an important public health burden in Southeast Asia and other endemic countries. Various genotyping methods have been applied to study the genetic variations of this human-restricted pathogen. Multilocus sequence typing (MLST) is one of the widely accepted methods, and recently, there is a growing interest in the re-application of MLST in the post-genomic era. In this study, we provide the global MLST distribution of *S*. Typhi utilizing both publicly available 1,826 *S*. Typhi genome sequences in addition to performing conventional MLST on *S*. Typhi strains isolated from various endemic regions spanning over a century. Our global MLST analysis confirms the predominance of two sequence types (ST1 and ST2) co-existing in the endemic regions. Interestingly, *S*. Typhi strains with ST8 are currently confined within the African continent. Comparative genomic analyses of ST8 and other rare STs with genomes of ST1/ST2 revealed unique mutations in important virulence genes such as *flhB*, *sipC*, and *tviD* that may explain the variations that differentiate between seemingly successful (widespread) and unsuccessful (poor dissemination) *S*. Typhi populations. Large scale whole-genome phylogeny demonstrated evidence of phylogeographical structuring and showed that ST8 may have diverged from the earlier ancestral population of ST1 and ST2, which later lost some of its fitness advantages, leading to poor worldwide dissemination. In response to the unprecedented increase in genomic data, this study demonstrates and highlights the utility of large-scale genome-based MLST as a quick and effective approach to narrow the scope of in-depth comparative genomic analysis and consequently provide new insights into the fine scale of pathogen evolution and population structure.

## Introduction

Typhoid fever poses a significant health threat to many endemic countries. *Salmonella enterica* serovar Typhi (*S*. Typhi), the etiologic agent, can be transmitted through contaminated food and water via the oral-fecal route. Annually, over 21 million cases and nearly 200, 000 deaths are reported worldwide (Crump and Mintz, 2010). Despite major treatment and prevention efforts, global typhoid cases remain very high (Lozano et al., 2010; Murray et al., 2010). The disease is also human restricted and the infected individuals could persist as long-term carriers, which in turn serve as the reservoir for new infections and outbreaks (Gonzalez-Escobedo et al., 2010).

The epidemiological investigation of *S*. Typhi is important for disease control such as during a disease outbreak to trace the potential sources. Over the last few decades, many molecular subtyping methods have been applied to genotype bacterial pathogens, and among these, multilocus sequence typing (MLST) is the most commonly used genotyping method to determine the ancestral lineages of many bacteria, including *S*. Typhi (Achtman et al., 2012; Leekitcharoenphon et al., 2012). This method allows discrete characterization of isolates using the internal fragments of housekeeping genes sequences (Urwin and Maiden, 2003; Achtman et al., 2012). The 7 loci MLST scheme of *S*. Typhi was first utilized by Kidgell et al. (2002) to determine *S*. Typhi lineages based on the known sequence types (STs). However, MLST is of limited use for monomorphic pathogen, such as *S*. Typhi, as their populations accrue very limited variations, thus hampering efforts in a population study. In the recent years, high-throughput WGS has become the ultimate approach to study bacterial population structure and phylogeny (Didelot et al., 2012; Chen et al., 2013; Sherry et al., 2013).

Based on MLST, presumably, the global most widespread *S*. Typhi is genetically characterized as ST1 and ST2 in the earlier studies (Kidgell et al., 2002; Zhang et al., 2011; Dahiya et al., 2013; Martínez-Gamboa et al., 2015). However, this conclusion was drawn from analyses of limited numbers of samples. There is also a lack of data on the emergence of uncommon STs which may have been circulating but remain undetected, nor is there a clear answer for the predominance of ST1 and ST2 globally. It is possible that these predominant and clonally related ST1 and ST2 have high transmissibility and/or enhanced virulence to circumvent killing by the innate immune system and probably acquired ability to evade host immune responses, allowing them to establish long-term carriage stage. To date, there are only a few uncommon STs reported for *S*. Typhi. These include ST8-typed isolate (422mar92 from Zaire, Africa, 1992) which was first reported by Kidgell et al. (2002) and ST3 (SARB64, Senegal, 1988; Hangzhou-31, Hang Zhou, China; Kidgell et al., 2002; Wang et al., 2009). Since then, there is no further record for ST8, at least from all available 7,089 *S. enterica* entries in the MLST database (last accessed 10 October 2015). Therefore, it is extremely challenging to gain full evolutionary insights, particularly in regards to virulence and pathogenesis of the pathogen, without first comparing the current widely disseminated populations with the seemingly “near-to-extinct” populations in the evolutionary timescales of *S*. Typhi; much less if these missing-link populations hardly exist or being identified. To test this hypothesis, we performed MLST on both local and global data, either experimentally through conventional MLST or WGS-derived, and scan for any uncommon STs, and to establish the population structure of this pathogen from the global perspective of *S*. Typhi population. Upon identification, the genomes of the uncommon STs were compared against the predominant ST1- and ST2-typed genomes (three reference genomes and six of our previously sequenced genomes). Other publicly available *S*. Typhi genomes were used as background comparison to provide an accurate framework to discern any unique variations carried by these rare populations. Our findings provide valuable information that is important for the understanding of the poor adaptation of *S*. Typhi with uncommon STs, which may otherwise be capable of disseminating globally.

## Materials and Methods

### Local and Regional Bacterial Strains Selection

To determine the STs of *S*. Typhi in this region, we have randomly selected 19 representative strains isolated from various clinical outcomes (outbreaks, sporadic cases, and human carriers). The *S*. Typhi strains were obtained over three decades (34 years; 1983–2008) from 13 distinct geographical locations in three endemic countries (Malaysia, Chile, and Papua New Guinea), which represent three large continents (Southeast Asia, Oceania, and South America). These strains include two each from Papua New Guinea and Chile while the remaining strains are from Malaysia. These strains have been previously described (Thong et al., 1995, 1996a,b, 2002; **Supplementary File 1**). Although the numbers of strains are relatively small, they were selected from the best available representatives from our collection. The bacterial cultures were further cryopreserved as glycerol stocks at -80°C in 50% glycerol for storage.

### Genomic DNA Extraction and MLST

Genomic DNA extracted from overnight cell cultures in Lysogenic Broth (Oxoid, Hampshire, UK) according to the manufacturer’s protocol (Promega Corporation, Madison, WI, USA) was used as PCR template. Housekeeping genes, *thrA* (aspartokinase+homoserine dehydrogenase*), purE* (phosphoribosylaminoimidazole carboxylase), *sucA* (alpha-ketoglutarate dehydrogenase), *hisD* (histidinol dehydrogenase), *aroC* (chorismate synthase), *hemD* (uroporphyrinogen III cosynthase), and *dnaN* (DNApolymerase III beta subunit) were targeted for the MLST scheme (Kidgell et al., 2002). PCR assays were carried out with ∼50 ng of DNA template, 150 μM (each) deoxynucleoside triphosphates, 1× PCR colorless buffer, 1.2 mM MgCl_2_, 0.2 μM of primer, and 0.5 U of Go Taq Flexi DNA Polymerase (Promega, Madison, WI, USA) in 25 μl reaction mixtures. PCR conditions used are as follows; initial denaturation at 94°C for 30 s, 30 cycles of denaturation at 95°C for 30 s, primer annealing at 55°C for 30 s, and extension at 76°C for 30 s; and a final extension at 75°C at 2 min. Reactions were performed using a PCR Master Cycler (Eppendorf AG, Hamburg, Germany). Products were separated by 1.5% agarose gel electrophoresis and visualized with GelRed (Biotium, Inc., Hayward, CA, USA) staining and UV illumination with a gel documentation system (Gel Doc 2000; Bio-Rad, Hercules, CA, USA). Primers used for the PCR assays are listed in **Supplementary File 2**. PCR products obtained were purified using PCR purification Kit (MEGAquick-spin^TM^, iNtRON Biotechnology, Seongnam, Korea) according to manufacturer’s instruction. The purified PCR products were submitted to a commercial sequencing facility for sequencing. Primers used for the sequencing reactions are listed in **Supplementary File 2**. The seven sequenced loci of 19 local and regional *S*. Typhi strains were first trimmed, edited and aligned using MEGA 6 (Tamura et al., 2013). Sequences were subsequently submitted to the MLST database^1^ and assigned existing or novel allele type numbers. The composite sequence STs were defined by the database based on the set of allelic profiles derived from each of the seven loci. Allele sequences for each strain were then concatenated to a final sequence length of 3,336 bp.

### Mlst using Whole Genome Sequences of Global Strains

To perform MLST for global strains, all accessible 1,814 whole genome sequences (WGSs) of *S*. Typhi (as of 21 September 2015) and 2 *S*. Paratyphi A strains and their background data were retrieved from NCBI Genome databases via anonymous file transfer protocol (FTP) at ftp://ftp.ncbi.nih.gov/genomes/ or manually downloaded from NCBI. The accession numbers of the reference, our previously sequenced, and published genomes are listed (**Supplementary Files 1** and **3**). The newly released genomes sequences, raw sequence data, and accession number can be accessed as a batch via European Nucleotide Archive accession number ERP001718 (Wong et al., 2015). The results of the ST assignment and unconfirmed allelic profiles were manually evaluated. In brief, all WGSs were first re-annotated and validated as previously described (Yap et al., 2012a,b) for standardization. The nucleotide sequences were then aligned against MLST database^1^ and assigned existing or novel allele type numbers. For validation, the WGSs were also submitted to Centre for Genomic Epidemiology MLST 1.8^2^ (Larsen et al., 2012) to assign ST based on the closest matches against the MLST databases. The composite STs (STs) were defined by the database based on the set of allelic profiles derived from each of the seven loci.

### MLST Phylogenetic and Data Analyses

All 1,783 concatenated sequences of MLST (local/regional strains and genome-derived; **Supplementary File 3**) with 100% allele recovery, including the allelic sequences of 2 outgroups sequences extracted from *S*. Paratyphi A complete genomes [*S*. Paratyphi A AKU_12601 (FM200053.1) and *S.* Paratyphi A ATCC 9150 (CP000026.1)] were aligned with MAFFT (Katoh and Standley, 2013) with parameter E-INS-i and a phylogenetic tree was constructed by approximate maximum-likelihood using FastTree2 with the generalized time-reversible model and Bayesian mixture model (GTR+CAT; Price et al., 2010). The statistical significance of phylogeny was estimated by bootstrap analysis with 1000 pseudoreplicates. Allele numbers and STs of this study were deposited in the publicly accessible *S. enterica* MLST database^1^. To determine the genetic polymorphism of *hemD* among *S. enterica*, all 333 *hemD* (864 bp) trimmed gene sequences were retrieved from MLST *S. enterica* database^1^. A multiple sequence alignment was then built with CLUSTALW in MEGA 6 (Tamura et al., 2013).

### Comparative Genomics Analysis of *S*. Typhi with Uncommon and Predominant STs

After the MLST analyses, the eight genomes subtyped with uncommon ST8 (76-1292, E01-5741, 05-8683, 206926, MDUST177, 627334), ST2233 (np45), ST2359 (ST821/98) were retrieved and studied in detail. The assembled genomes were annotated and mapped to the reference genomes (CT18) as previously described (Ho et al., 2012; Yap et al., 2012a). The tRNA and tmRNA were predicted using Aragorn (Laslett and Canback, 2004) whereas the rRNA was predicted with rRNAMMer (Lagesen et al., 2007), and manually validated as described earlier (Osama et al., 2012; Suhaimi et al., 2014). To compare, we have included three reference genomes and six of our own published sequenced genomes (described earlier), representing ST1 (Ty2, P-stx-12, Ty21a, BL196, CR0044) and ST2 populations (CT18, CR0063, UJ308A, UJ816A, ST0208). The pan-genome data of these genomes was obtained using PGAP (Zhao et al., 2012) and compared as previously described (Yap et al., 2014). The phage regions of the pan-genomes identified were predicted and confirmed with web server PHAST (Phage Search Tool; Zhou et al., 2011) and intact phage regions were manually examined. For plasmid analyses, the plasmid replicons were detected using PlasmidFinder 1.3 database (Carattoli et al., 2014) and plasmid MLST was determined using pMLST1.4 (Carattoli et al., 2014) and validated with manual inspection. The acquired antimicrobial genes were identified and determined using ResFinder 2.1 (Zankari et al., 2012). To understand other possible genetic events that shaped the rare STs populations, we performed CRISPRs (clustered regularly interspaced short palindromic repeats) sequences and Restriction–Modification (RM) system prediction analyses using CRISPRFinder (Grissa et al., 2007) and Restriction-ModificationFinder1.1^3^, respectively. For virulence genes and *Salmonella* Pathogenicity Islands (SPIs) analyses, the annotated sequences were mapped against Virulence Factor Database (VFDB) and Pathogenicity Island Database (PAIDB) and KEGG pathway (Chelvam et al., 2015), respectively using BLASTn (>98% identity and 60% coverage, E-value < 1x^-10^) and Artemis (Carver et al., 2008).

### Robust Phylogenomic Analyses using 1,808 Global *S*. Typhi Genomes

Phylogenomic analyses were conducted to better understand the phylogeny of the uncommon STs in the perspective of all *S*. Typhi genomes. To compute large genome samples is computationally demanding, time-consuming and technically challenging. Therefore, we utilized a newer approach of alignment-free algorithm using andi v1.4 (Haubold et al., 2015) to rapidly compute large-scale evolutionary distances between 1,808 *S*. Typhi (∼8.6 Gbases; six genomes were excluded from phylogenetic analyses due to low quality) and bias from mapping against reference genome can be avoided. The approach based on a new distance measure, *d*_a_, which approximates local alignments by anchoring them with long, unique matches of a minimal length. The matches are equidistant in the query and the subject is equivalent to restricting the analysis to ungapped alignments. These exact matches were then searched efficiently using enhanced suffix arrays. The anchor distances and arrays were computed using the multithreaded UNIX command-line in the andi v1.4 package (Haubold et al., 2015). The resulting data was aligned, analyzed, transformed and the phylogenetic tree (NJ method) was constructed with SplitsTree4.12.6 (Huson and Bryant, 2005). A rooted tree was inferred using the *S*. Paratyphi A AKU 12601 and *S*. Paratyphi A ATCC 9150 as outgroup. The approach was repeated for 1,806 genomes without the outgroup to generate an unrooted tree. To enhance the visualization of the gigantic tree, the tree was re-rooted with an *S*. Typhi strain from the earliest *S*. Typhi cluster to the outgroup. The node to *S*. Paratyphi A was also collapsed (very long branch length) to improve visualization of the massive tree.

## Results

### High Allele Recovery Rate in Currently Sequenced *S*. Typhi Genomes and STs Identification from Both Global and Local/Regional Data

Composite allelic profiles were successfully recovered from 1,762 (97%) *S*. Typhi genomes with 100% sequence coverage and 43 (2.3%) genomes using the top BLAST hits criteria (>99% sequence coverage, E-value cut-off <1 × 10^-10^). The remaining profiles were predicted using the top BLAST hits criteria (coverage less than 99%) against MLST database, which accounted for only less than 0.5% of the genomes studied. Out of the 1,827 global and local/regional strains, 1282 (70.2%) and 536 (29.3%) were subtyped as ST1 and ST2, respectively (one unknown ST strain; **Supplementary File 3**). Our findings showed that ST1 and ST2 have been homogenously circulating for over the period of 109 years and represent the current predominant populations globally. Both STs were detected as early as in the year 1905, the oldest strain included in our study. Likewise, our local/regional strains exhibited the same pattern of ST1/2 dominance throughout a period of 30 years. We also attempted to detect the geographical structuring of both STs in three levels (continents, region within continents and country), but a very limited evidence was found except for the archipelago of Samoa (116 ST1 and 1 ST2) and Papua New Guinea (1 ST1, 48 ST2) of the Oceania regions, in which an obvious skew toward one ST was observed. We also found no association between STs and source of isolations in both global and local/regional collection. Interestingly, only 6 strains (76-1292, E01-5741, 05-8683, 206926, MDUST177, 627334) were subtyped as ST8, a rare ST but common in the African regions (Central, North, and South Africa). Remarkably, ST8 was undetected outside of African continent for more than a century. Although, this population was geographically-restricted and seemingly unsuccessful in its dissemination to other parts of the world, the latest ST8-typed strain identified in our study was dated 2012. Since the oldest strain was recovered as earlier as the year 1976, hence, these strains have been circulating in the community for at least 37 years, indicating an establishment of the long-term local reservoir in the African region. It is challenging to confirm whether or not this population has long existed before the aforementioned periods, or represents the ancestral population to ST1 and ST2. Nevertheless, the containment of ST8-typed population only within the African continent but nowhere else is consistent with the notion of the early migration of humans out of Africa, and thus the spread of the pathogens, as proposed by Kidgell et al. (2002). As the bacterium accrues very limited variation, it is highly possible that these two populations (ST1/ST2 and ST8) shared a very recent common ancestor which later diverged into two distinct populations. Apart from ST8, we also detected two uncommon STs, ST2233 (np45) and ST2359 (ST821/98) from South Asia (Nepal) and South America (Argentina), respectively.

### High Sequence Conservation of *S*. Typhi from Various Endemic Regions of Typhoid

**FIGURE 1:**
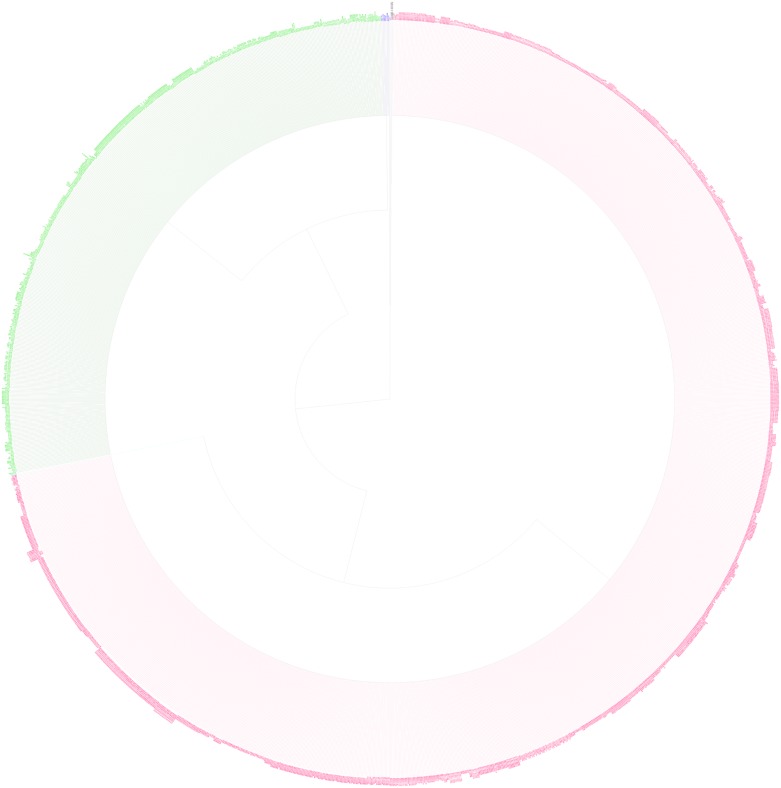
**Maximum Likelihood tree shows the genetic relationships of 3,336 bp concatenated MLST genes sequences derived from the seven housekeeping loci of 1,783 *S*. Typhi strains isolated globally.** The tree is rooted with *S*. Paratyphi A. The colors of the strains label represent the STs; red for ST1, green for ST2, blue for ST8, pink for ST2233, light blue for 2359, and black for outgroup. All branches have more than 90% support.

All seven MLST loci were successfully recovered for the 1,781 *S*. Typhi strains that were obtained from diverse geographical regions (7 continents, 65 countries) and sources of isolation (blood, stool, urine, and environment) over a period of 109 years. The sizes of the trimmed sequenced alleles of all seven loci ranged from 399 to 501 bp. The concatenated sequence amounted to 3336 bp, an approximate 0.07% of the size of *S*. Typhi complete genome (with reference to the CT18 complete genome). The pairwise allelic alignments showed that the average sequence divergence of these loci was very low, which were contributed mainly by *hemD*, which delineates two large populations (ST1 and ST2). The *hemD* gene exhibited only two allelic profiles, 1 and 2 (single synonymous nucleotide polymorphism). The SNP of the *hemD* occurs at position 129 of the trimmed partial sequence where the thymine base of *hemD*1 is replaced by cytosine in *hemD*2. Further investigation on the diversity of *hemD* genes by including a total of 333 *hemD* genes from *S. enterica* (retrieved from MLST database^4^ revealed that the polymorphic site (C129T) of the *hemD* is conserved across all members of the group, except for the polymorphism carried by *hemD*1 of *S*. Typhi (**Supplementary File 4**). Our global phylogenetic analysis of *hemD* gene from *Enterobacteriaceae* members indicated that the *hemD*1 allele may have diverged simultaneously or later from the MRCA, given the fact that the *hemD*2 allele is conserved throughout the *Salmonella* sp. and its closely related species, *Escherichia coli* and *Shigella* sp. Although, polymorphisms were also detected in *hisD*, *thrA*, and *dnaN* of the uncommon STs (ST 8, ST2233, ST2359), but these allelic frequencies were extremely low, in which they differed by only one SNP each (ST8-*hisD*1 to *hisD*3; ST2233-*dnaN*1 to *dnaN*478; ST2359-*thrA*5 to *thrA*545). To understand the phylogeny of these STs, an MLST phylogenetic tree of all the *S*. Typhi strains was inferred using the maximum-likelihood approach from the concatenated sequences. We observed two main clonal clade harboring large numbers of ST1 and ST2 strains, which appeared to be a very recent divergence from its common ancestor. The ST8-typed population is phylogenetically close to ST2, whereas ST2359 forms a polytomy with ST1. However, the node containing ST2233 that is closer to the root is polytomically unresolved from ST1/ST2 clades. Although the bifurcation of ST1/ST2 may be the result of the addition of ST2233-typed strain, but the internal nodes of ST8 and ST2359 remain unresolved, most likely the result of short divergence and/or low discriminatory power of MLST (**Figure 1**). Such clonal relationships would be expected for a recent bottleneck that allowed only a few clones to survive, possibly through purifying selection, such as those commonly observed in other monomorphic bacteria.

### Phylogenomic Analyses Revealed Strong Local Phylogeographical Signals

**FIGURE 2:**
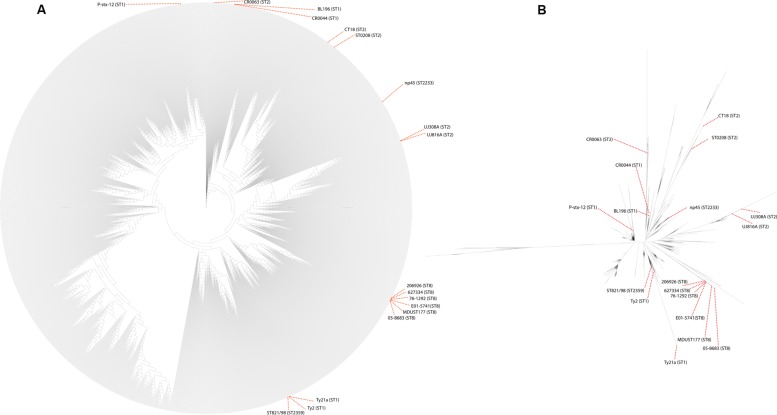
**Alignment free-based phylogenomic tree generated from 1,806 *S*. Typhi and 2 *S*. Paratyphi A genomes.** All reference, our previously sequenced and the uncommon ST-typed genomes with their STs were labeled. Other strain labels were removed to enhance visualization. **(A)** The phylogenomic tree was rooted with *S*. Paratyphi A. The node to *S*. Paratyphi was collapsed (very long branch length) to enhance visualization of the large tree. **(B)** Unrooted tree generated from 1,806 *S*. Typhi genomes.

To view the phylogenetic relationship of these strains with much higher resolution, a whole-genome based phylogenetic tree was built from 1,808 *S*. Typhi genomes (**Figure 2**). This robust phylogenomic tree shows that all *S*. Typhi is monophyletic and distinct from its closest counterpart, *S*. Paratyphi A with long branch length, reiterating the classical phylogeny of these two serovars, which diverged from each other earlier through convergent evolution (Holt et al., 2009). In this phylogeny, there is a clear separation from early diverging basal groups and the core groups that diverge later. Remarkably, these earliest basal groups closest to the root consisting of *S*. Paratyphi A are almost entirely isolated from Africa, which supporting the “theory of human-pathogen co-evolution” since the early human migration out of Africa. Moving further from the roots, the pathogen seems to have widely propagated across various regions, evidenced by several sub-clusters isolated from Africa, South America, Asia, and Australia/Oceania. The frequencies of these basal groups are relatively low, suggesting that these groups may have been under-sampled or reflect true disappearance, plausibly as a result of purifying selection. In contrast, the more recent core clade experienced a radiation that resulted in at least three large clusters with many sub-clusters observed. Interestingly, we detected strong signals of phylogeographical structuring in which some sub-clusters were recovered from the same geographical regions, despite being separated for years, highlighting the importance of local reservoir in maintaining endemicity in those regions. This observation corroborates our earlier study (Yap et al., 2014) in which a human carrier strain (CR0044-isolated from a food handler) was indeed highly related to the outbreak strain (BL196-isolated from a large outbreak in Malaysia), emphasizing the propensity of human carriers to trigger future outbreaks in the endemic regions. Notably, the ST8-typed population appeared to be ancestrally related to the African and Asia sub-cluster, in which it diverged from the common ancestor to form a distinct terminal cluster. Interestingly, it was observed that ST8-typed population shared the MRCA with a sub-cluster exclusive to Australia/Oceania strains, suggesting that clonally related local strains are circulating in the regions. In contrast, the ST2359-typed ST821/98 was found highly related to the reference genome Ty2 (isolated from Russia) and its derivative vaccine strain Ty21a. Other strains in the sub-cluster of ST821/98 were in fact geographically related, such as those few isolated from Russia and Europe. To note, the close relative of ST821/98; the attenuated Ty21a and its parental strain, Ty2 were both reported to share the same genetic mutant that partly contribute to attenuation of the live vaccine. The ST2230-typed np45 strain was in the major cluster I together with the CT18 and ST0208 (isolated from Southeast Asia). The sub-cluster in which np45 located was geographically restraint to mainly South Asian regions (India and Bangladesh). This sub-cluster was ancestrally related to some other smaller clusters that contain uniformly Asian and African strains, to some extent, indicative of population displacement by the relatively recent clones. In comparing the major clusters of the phylogeny, the strains were separated by such short internal branch length that they appeared to have diverged nearly simultaneously. Most of the sub-clusters are separated by an extremely shallow branch, indicating shared common ancestors, and apparently were quite successful in its dissemination, illustrated by its appearance over several continents. Notably, few strains from the Australia/Oceania regions were detected in the terminal of the relatively longer branching of the deep node, suggesting divergence of variants from the existing nodes, perhaps arose in response to intense selection pressures.

### Comparative Genomics of Rare STs Strains Revealed Unique Gene Signatures

Because of the limited dissemination of the rare STs, the ST8-typed strains, in particular, which are not found in other countries outside the African region, we attempted to elucidate the genomic signatures, which might present potential key factors contributing to their poor dissemination. The differences between the seemingly unsuccessful and successful population may explain the evolutionary events that are required for successful infection, transmission, and/or maintenance of carriage state. As the reservoir of *S*. Typhi is mainly human carrier, the low prevalence of this rare STs may reflect its poor adaptation in the host (carriage state), which is required for wide dissemination. This postulation is supported by the fact that the ST 8-typed population was not identified outside of African region, consistent with the route of early human migration out of Africa. In contrast, although ST2233 and ST2359 were rarely identified but their presence outside of African region may reflect new emerging variants. As the current knowledge of S. Typhi is solely based on the predominant STs, therefore, we attempted to perform comparative genomics to dissect any genetic signatures of this important population. In terms of basic genomic features, the genome sizes of ST8-, ST2233-, and ST2359-typed isolates ranged from 4.69 to 4.85 Mb with an average GC content of 52.1%. The CDS contents of these genomes ranged from 4839 to 5046 with an average tRNA and rRNA of 71 and 5, respectively. Every genome also carries one tmRNA each. The basic genetic features of these genomes are similar to many previously described genomes (Parkhill et al., 2001; Deng et al., 2004; Holt et al., 2008). High genomic synteny and conservation, with limited evidence of recombination, were also observed in the uncommon STs-typed strains, which shared similar findings with other previous studies (Holt et al., 2008; Yap et al., 2014).

*Salmonella* Pathogenicity Islands are unique pathogenicity regions with high numbers of virulence factors (VFs) carried by *S. enterica*, which facilitate the bacterium in host colonization, invasion and maintenance of virulence in the host (Coburn et al., 2007; Dougan and Baker, 2014). SPI-7, the unique virulence region of *S*. Typhi was found intact in all uncommon ST-typed strains. Further, all the strains commonly shared the 227 out of 242 VFs compared. The remaining VFs were shared by at least two genomes except for the strain 76-1292, which carry a unique gene, *astA*, which encode for heat stable enterotoxin. All the compared genomes carried the hypothetical virulence gene, *t0576*, which was absent in CT18. Interestingly, the *tsaC* was only present in the ST8-typed strains and CT18 but lacks in other genomes. Five out of six genomes of ST8-typed strain lack *pilV* but carry the *pilV2*, an alternate and duplicated gene of *pilV*. Notably, four out of six ST 8-typed genomes carried a *seD* gene which was absent in all other genomes. On the contrary, *STY2517* was the only unique gene present in CT18 but absent in others. Notably, Np45 genome which was subtyped as ST2233 lacks VFs needed for secretion machineries such as *sciT*, *ssaV*, and *sthB* although other virulence genes in the respective operons were fully intact, which likely indicates a true absence rather than sequencing artifacts. The VFs with the highest variation were noted in an outer fimbriae gene, *sefC*, although other VFs are highly conserved.

Interestingly, we detected variations in several essential genes, which are involved in virulence and pathogenesis. Synonymous point mutations were identified in three virulence genes; *flgE* (unique SNP), *pipB* (non-unique SNP), *spaO* (non-unique SNP). We also identified shared non-synonymous point mutations in molecular chaperone *clpB* (C338R), fimbriae-like periplasmic protein *Sfm/fimF* (V64A), E3 ubiquitin–protein ligase/*sseJ* (K713E), *hilD* (K209E) and *tviE* (K266N), although not unique to six of the ST8-typed genomes but was identified only in less than 2% of the 1,808 genomes studied. Notably, we also discovered that all ST8-typed genomes shared the identical unique non-synonymous point mutations in three important virulence genes, the *flhB* (E378G), *sipC* (G176S) and *tviD* (R209Q), which were not identified in all other ST1- and ST2-typed populations. In addition, deletions were also detected in two virulence genes, *fimI* (8 bp deletion) and pseudogene *misL* (15 bp deletion), which may lead to a frameshift and premature stop codon. However, we found no unique SNPs of VFs among all eight genomes, except for the *tviD* which was shared by ST821/98 (ST2359), suggesting that they may have undergone different evolutionary paths.

### Antimicrobial Resistance Genes Analyses

Of all six ST8-typed strains that were obtained from the African region, only two (76-1292 and 05-8683) acquired antimicrobial resistance genes. In fact, both the 76-1292 and 05-8683 are predicted multidrug-resistance. The 76-1292 strain harbors *sul1* (sulphonamide resistance), *aadA1* (aminoglycoside resistance), *blaOXA-1*, *catA1* (phenicol resistance) and *tetA* (tetracycline resistance) genes, which were found on IncI1 (pMLST 186) and IncP plasmids. In contrast, 05-8683 carried a rare *ereA*, a gene that is associated with azithromycin resistance (macrolide), apart from other common antimicrobial genes of *catA1* (phenicol resistance), *sul1* (sulphonamide resistance), *tetB* (tetracycline resistance), *dfrA5* (trimethoprim), *blaTEM-1B* (beta-lactam), which were found located in a multireplicon IncF plasmids with pMLST of [F1:A-:B49]. However, both 76-1292 and 05-8683 lacks the IncHI1 plasmid, a replicon type which is commonly associated with the widely disseminated H58-haplotyped *S*. Typhi (Wong et al., 2015). On the contrary, no acquired antimicrobial genes were identified for np45 (ST2233) and ST821/98 (ST2359) strains.

### Limited Evidence of Lesion in CRISPRs, Phages, and Restriction–Modification (RM) System

Phage elements were thought to be one the main driving forces of evolution in *S*. Typhi and *S*. *enterica* (Holt et al., 2008); however, we detected limited evidence of novel intact phages in all eight ST8-, ST2230-, and ST2359-typed strains. In fact, the shared eight regions are highly similar with only slight sequence variations (attributed mainly by the gain/loss of hypothetical and phage structural genes), as identified in the reference genomes. The CRISPRs, a known prokaryotic adaptive immune system that resists invading nucleic acids, may cause DNA mutations and damage (Li et al., 2014). In our analysis, CRISPR spacers and length, direct repeat length, and consensus sequence were commonly shared among genomes, with very little to no variation. We detected an identical six spacers with direct repeats length of 29 bp and with three variants (385, 394, or 420 bp) of CRISPR length. Although, CRISPR has been found to be correlated with STs in some study for subtyping, at least for *S. enterica* (Fabre et al., 2012), we found very limited evidence of variation in *S*. Typhi to be of valuable use in subtyping using CRISPRs. Besides, the RM systems represent known barriers against the entry of foreign DNA. Yet, the RM system is poorly understood in *S*. Typhi. In this study, we revealed extensive repertoires of RM systems, which contain four predicted RM systems (Type I, Type II, Type III, and Type IV) in all studied genomes. The Type I system of *S*. Typhi, which include genes encoding methyltransferase (*M.SptAIII*) and specificity subunits (*S.SptAIII*) were highly similar to that of *S*. Paratyphi A ATCC9150, whereas *EcoKI*, which codes for restriction enzyme is homologous to *E. coli* K-12. The genes (*M.SenAboDcm*, *M.SenSPBIII*, *M.Sen158III*) in Type II code for methyltransferases, which are commonly identified in various *S. enterica*. Type III has both the restriction and methylation domains; the genes *SenAZII* (Restriction enzyme) and *M.SenSPBII* (methyltransferase) were of homologs of *S. enterica* subsp. arizonae serovar 62:z4,z23 and *S*. Paratyphi B SPB7, respectively. For Type IV, only *StyLT2Mrr* was identified, which involves in methyl-directed restriction as carried by the closely related *S*. Typhimurium LT2. The comparative genomics data demonstrated that the RM systems were intact with high similarities, suggesting no functional lesions. Notably, two and only plasmid-bearing ST8-typed *S*. Typhi strains (05-8683 and 76-1292) carried an additional Type 1 *EcoR124I* encoding restriction enzyme (*E. coli*) and Type II *M.EcoGIX* encoding methyltransferase (*E. coli* O104: H4 str. C227-11), highlighting the potential role of RM systems in plasmid acquisition.

## Discussion

In the present study, our MLST data collection for *S*. Typhi, to date, is the largest in numbers and were from the most diverse representatives available (geographical regions, sources) over a period of 108 years. We successfully demonstrated the practicality of scanning 1000s of bacterial genomes. With this scale of data, we also demonstrated unequivocally that ST1 and ST2 are the two most predominant STs globally and are highly successful in dissemination since their emergence. Our results concurred with previous analyses, which relied on limited numbers of *S*. Typhi strains (Kidgell et al., 2002; Zhang et al., 2011; Dahiya et al., 2013). The populations of ST1 and ST2 have been expanding drastically since their emergence from the MRCA and are still co-circulating in the population for at least a century.

*Salmonella* Typhi, a human-restricted pathogen has long been associated with human migration as well as co-evolution with humans (Kidgell et al., 2002). In this study, no evidence of bias in the spatial and temporal distribution of STs was identified from the MLST data, suggesting that the rates of human migrations across continents have been extremely high to disseminate equal genotypes across the human populations. The *S*. Typhi population may have suffered a recent bottleneck during its course of evolution, evidenced by the limited STs and loci recombination. However, the evolutionary signals from the MLST alone are also rather weak, probably owing to its short evolutionary distance which supports the previous age estimates (∼50,000 years-old; Kidgell et al., 2002) of this relatively “young pathogen.” Similar observations have been observed in other monomorphic pathogens such as *Mycobacterium tuberculosis*, the etiologic agent of tuberculosis (Achtman, 2008; Gagneux, 2012).

It is interesting to note that the “primitive” ST3 was completely lost in the population (at least from our data) since the first report of its emergence in Africa in Kidgell et al. (2002). The enigmatic disappearance of this ST, perhaps due to the transient Darwinian selection that rendered the bacteria less fit as a result of harmful mutations accumulated over evolutionary time. However, we cannot rule out the possibility of genetic drift or/and the effects of environmental changes such as those observed in its close relative, *S*. Paratyphi (Zhou et al., 2014). Indeed, the understanding of such evolutionary dynamics is lacking without constructing a genealogy of *S*. Typhi with the inclusion of all populations over a long period of time.

Whilst the identification of uncommon STs were reported intermittently, such as ST890 and ST892 in China (Zhang et al., 2011) and ST1856 in Indonesia recently, these events are extremely rare, and possibly reflects the variants from the existing clones. A similar phenomenon was also observed for ST2233 and ST2259 in this study. On the contrary, the population of ST8 is likely to have poor dissemination and adaptation in humans, in view of its low prevalence globally, as well as being geographically-restricted. It is reasonable to speculate that, the population may still maintain the ability to cause human carriage, perhaps poorly, given the fact that the population has been around for at least 37 years. This was further supported by the WGS phylogenomic analyses, which revealed that the ST8 population is likely to diverge from the shared common ancestors of ST1- and ST2-typed population, before substantially diluted by the expansion of ST1 and ST2-typed *S*. Typhi out of Africa.

The findings from the comparative analyses of the ST8-, ST2230-, and ST2359-typed genomes relative to ST1- and ST2-typed genomes could be the key factors underpinning the successful traits of predominant populations. As we attempted to interrogate the genomic signatures of these genomes, we found very limited evidence of marked variations in regards to gene gains/loss. The SPI-7, which is implicated as the main virulence repertoires of *S*. Typhi, was completely intact in all *S*. Typhi with uncommon STs, in addition to other SPIs. This finding is consistent with an earlier study that *S*. Typhi bearing SPI-7 was less invasive than the one with the entire SPI-7 missing (Bueno et al., 2004), suggesting that the presence of SPI-7 may be relevant for the fitness of the bacterium. Besides, the genomes harbor very limited numbers of strain-specific genes, which are mainly hypothetical or phages-related. A similar observation was also noted in phage regions, whereby the sequences are highly conserved with little to no variations, except for the few variations punctuated in between the intact phage regions, suggesting the possible hotspots for genome variations. Similarly, the conservation of CRISPRs regions in *S*. Typhi of different STs detected may reflect common defense mechanism acquired by the isolates to fight off incoming foreign DNA. The abovementioned findings are of little surprise as this clonality is well documented in previous studies (Roumagnac et al., 2006; Holt et al., 2008). To probe further, we performed RM system analyses, to detect for any genomic peculiarity of this population. Although, our data indicated no lesion detected in the RM systems; however it is worth mentioning that both the plasmid bearing genomes carried an additional RM systems in comparison with other studied genomes. The presence of these RM systems may facilitate the intake of plasmids/acquired resistance. Such phenomenon has been observed in various bacteria (Oliveira et al., 2014), but its function in *S*. Typhi is so far, remains poorly understood, particularly its role in moderating virulence of the pathogen in the human host. The presence of plasmids in the 76-1292 and 05-8683, confer these strains with MDR phenotype, which may enhance the virulence of these strains. Nonetheless, these plasmids may grant little fitness advantage since the ST8-typed population with the MDR traits were missing in the subsequent isolations. It is likely that the presence of plasmids for maintaining the MDR traits may incur additional fitness burden on the already-less-fit bacterium, thus impacting the rate of successful infection and transmission. Such notion is also implicated in some *S*. Typhi population in which the plasmids were chromosomally integrated (Wong et al., 2015), possibly in the similar efforts to reduce fitness burden.

Intriguingly, our analyses indicated that the salient differences between the ST8- and ST1/ST2-typed populations are the presence of several non-synonymous mutations in few key virulence genes, such as the *flhB*, *sipC*, and *tviD*, which are uniquely present within this ST8-typed population. The affected gene, *sipC*, which encodes for a type III effector protein of the SPI-1 involved in translocation and active modulation, which has been implicated in host invasion. Previous studies have demonstrated that *Salmonella* mutant strains lacking the *sipC* were less invasive (Myeni and Zhou, 2010). An earlier study of *flhB* in *S*. Typhimurium showed that the mutation in the gene (structural gene for hook-associated protein 1; HAP1) resulted in polyhooks and altered flagellar hook length, which may have a severe impact on motility and adhesion of the bacterium (Hirano et al., 1994). The virulence of *S*. Typhi often correlates well with the presence of the Vi antigen (Arricau et al., 1998; Wetter et al., 2012). The mutation found in *tviD*, which is required for biogenesis of the Vi polysaccharide, may potentially affect the virulence of the pathogen.

The genomes of ST8 also harbor a numbers of non-synonymous mutations in several virulence genes (*clpB*, *Sfm/fimF*, *sseJ*, *hilD*, *tviE*) which are involved in invasion, colonization, virulence and regulation in various stages of pathogenesis. Although, these genes are not uniquely acquired, but may be related to the transitionary mutations occur along the evolutionary timeline in gaining fitness advantages. Notwithstanding, it is important to note that although ST1 and ST2 are seemingly successful, but the extent of its fitness remains unclear. The mutations may be accumulative rather than occurring at a single event since some of these mutations were strain-specific, but others are commonly shared unique mutations. Further, loss of function by premature stop codon or truncation from deletion/insertion in the virulence genes of *S*. Typhi is quite rare but the deletion in *fimI* and *misL* may suggest a selective advantage gained for such aberration. The deletion in *fimI*, a gene in type I fimbrial operon, may affect the biofilm formation ability of *S*. Typhi, as reported in some studies of the closest model, the *S*. Typhimurium (Teplitski et al., 2006). The *misL* (located at SPI-3) gene, which is an important colonization factor in the intestinal persistence during the infection of *S*. Typhimurium was disrupted by deletions. Although some essential genes such as *misL*, may be annotated as a pseudogene, the functionality of these pseudogenes in *S*. Typhi remains unclear. For example, *shdA* which was annotated as a pseudogene, was recently found to be functioning in *S*. Typhi (Urrutia et al., 2014), thus the effect of pseudogenization remains speculative. Interestingly, a very limited number of mutations from ST8-typed *S*. Typhi were shared with np45 and ST821/98, indicated that these populations were not closely related, and apparently, they evolved under distinct evolutionary events. These findings are in line with the phylogenomic analyses and MLST data, in which these strains were distantly related to ST8-population, suggestive of variants from the existing clones. However, we have very limited evidence to imply for its poor dissemination and whether or not this population is newly emerged, re-emerging or on the verge of “extinction,” explained largely on the basis of intermittent and short encounters, limited numbers of samples available and phylogeographical incongruence.

It remains elusive as to why strains of ST1 and ST2 are evolutionarily selected, albeit *S*. Typhi is thought to be shaped by weak diversifying selection forces along its evolutionary timeline (Roumagnac et al., 2006; Holt et al., 2008). It is reasonable to speculate that these successful STs may have predisposed to advantageous genetic variations that drive evolutions, allowing the pathogens to be more adapted in the host as observed hitherto in several genomic studies of *S*. Typhi (Parkhill et al., 2001; Deng et al., 2004; Baddam et al., 2012a,b; Ong et al., 2012; Yap et al., 2014; Hendriksen et al., 2015). Thus, extensive genomic studies of the *S*. Typhi strains from the endemic regions, particularly with the inclusion of rare subtypes of *S*. Typhi are required to construct full-scale evolutionary history to understand the mutational events that have occurred along the evolutionary timeline.

In summary, although the *S*. Typhi strains were obtained from diverse geographical locations spanning over a century, extremely low divergence was observed with two predominant STs (ST1 and ST2) being identified, a finding that was unequivocally supported by both analyses (experimentally and genome-derived STs). From the large scale scanning, we have identified three highly uncommon STs, the ST8, ST2233, and ST2359. Notably, the ST3 together with two recently reported novel STs (ST890 and ST892) identified in the previous studies are completely absent. The present findings provided strong evidence that *S*. Typhi strains possess a high level of temporal stability and phylogeographical structuring, supported largely by the phylogeographical signals observed in the phylogenomic tree. While many of the genome features have been studied earlier, this work highlighted the genomic signatures of the as-yet-uncharacterized ST8-typed population, which exhibit “unsuccessful trait” that may play vital roles in their poor dissemination. The understanding of these traits may have an important impact on disease control and vaccine development as this population may reflect models of attenuation.

## Author Contributions

K-PY designed the study, performed the experiment and analyses, and wrote the manuscript. K-PY and WH interpreted data. K-PY, WH, HG, LC, and KT assisted in data interpretation, drafting, and critically reviewed the manuscript and contributed important intellectual input. KT provided funding for the project and supplied equipment and research consumables. KT and LC supervised the project. All authors contributed to the editing of the manuscript and all authors read and approved the final manuscript.

## Conflict of Interest Statement

The authors declare that the research was conducted in the absence of any commercial or financial relationships that could be construed as a potential conflict of interest.

## Footnotes

1 http://mlst.warwick.ac.uk

2 https://cge.cbs.dtu.dk/services/MLST/

3 https://cge.cbs.dtu.dk/services/Restriction-ModificationFinder/

4 http://mlst.warwick.ac.uk
