# Supplementary material for: Global MLST of Salmonella Typhi Revisited in Post-genomic Era: Genetic Conservation, Population Structure, and Comparative Genomics of Rare Sequence Types
Source: Front Microbiol. 2016 Mar 2;7:270. doi: 10.3389/fmicb.2016.00270 (PMC4774407; doi:10.3389/fmicb.2016.00270)
Supplement: Supplementary file 4 [file Data_Sheet_4.PDF]

Additional File 3

	540–589
S. Typhi Ty2 hemD	TATTTCTCGATTGGCCGCACTACGGCGCTCGCCCTTCATACCGTTAGCGG
S. Typhi CT18 hemD	TATTTCTCGATTGGCCGCACCACGGCGCTCGCCCTTCATACCGTTAGCGG
S. Montevideo 507440-20 hemD	TATTTCGCGATTGGCCGCACCACGGCGCTCGCCCTTCATACCGTTAGCGG
S. Tennessee TXSC08-19 hemD	TATTTCGCGATTGGCCGCACCACGGCGCTCGCCCTTCATACCGTTAGCGG
S. Paratyphi A AKU_12601 hemD	TATTTCGCGATTGGCCGCACCACGGCGCTCGCCCTTCATACCGTTAGCGG
S. Enteritidis SEJ hemD	TATTTCGCGATTGGCCGCACCACGGCGCTCGCCCTTCATACCGTTAGCGG
S. Dublin hemD	TATTTCGCGATTGGCCGCACCACGGCGCTCGCCCTTCATACCGTTAGCGG
S. Abony 0014 hemD	TATTTCGCGATTGGCCGCACCACGGCGCTCGCCCTTCATACCGTTAGCGG
S. Agona 24249 hemD	TATTTCGCGATTGGCCGCACCACGGCGCTCGCCCTTCATACCGTTAGCGG
S. Thompson RM6836 hemD	TATTTCGCGATTGGCCGCACCACGGCGCTCGCCCTTCATACCGTTAGCGG
S. Gallinarum/pullorum CDC1983-67 hemD	TATTTCGCGATTGGCCGCACCACGGCGCTCGCCCTTCATACCGTTAGCGG
S. Javiana CFSAN001992 hemD	TATTTCGCGATTGGCCGCACCACGGCGCTCGCCCTTCATACCGTTAGCGG
S. Gallinarum/pullorum RKS5078 hemD	TATTTCGCGATTGGCCGCACCACGGCGCTCGCCCTTCATACCGTTAGCGG
S. Weltevreden 2007-60-3289-1 hemD	TATTTCGCGATTGGCCGCACCACGGCGCTCGCCCTTCATACCGTTAGCGG
S. Paratyphi BSARA61 hemD	TATTTCGCGATTGGCCGCACCACGGCGCTCGCCCTTCATACCGTTAGCGG
S. Saintpaul SARA24 hemD	TATTTCGCGATTGGCCGCACCACGGCGCTCGCCCTTCATACCGTTAGCGG
S. Bredeney CFSAN001080 hemD	TATTTCGCGATTGGCCGCACCACGGCGCTCGCCCTTCATACCGTTAGCGG
S. Abaetetuba ATCC35640 hemD	TATTTCGCGATTGGCCGCACCACGGCGCTCGCCCTTCATACCGTTAGCGG
S. Heidelberg CFSAN002069 hemD	TATTTCGCGATTGGCCGCACCACGGCGCTCGCCCTTCATACCGTTAGCGG
S. Cubana CFSAN002050 hemD	TATTTCGCGATTGGCCGCACCACGGCGCTCGCCCTTCATACCGTTAGCGG
S. Schwarzengrund CVM19633 hemD	TATTTCGCGATTGGCCGCACCACGGCGCTCGCCCTTCATACCGTTAGCGG
S. Bareilly CFSAN000189 hemD	TATTTCGCGATTGGCCGCACCACGGCGCTCGCCCTTCATACCGTTAGCGG
S. Muenchen SARA7 hemD	TATTTCGCGATTGGCCGCACCACGGCGCTCGCCCTTCATACCGTTAGCGG
Shigella flexneri 2a 301 hemD	TATTTCGCCATTGGACGCACCACCGCACTGGCGCTACATACCGTAAGCGG
Escherichia coli ATCC25922 hemD	TATTTCGCCATTGGACGCACCACCGCGCTGGCACTACATACCGTAAGCGG
consensus	******.*.*****.*****.**.**.**.**.**.********.*****
